# Prise en charge médico-chirurgicale des mycétomes à l'hôpital Somine Dolo de Mopti (Mali)

**DOI:** 10.48327/mtsi.2021.170

**Published:** 2021-11-10

**Authors:** Terna TRAORE, Layes TOURE, Mathias DIASSANA, Mamadou NIANG, Emmanuel BALLO, Boubacar S COULIBALY, Aristote HANS-MOEVI

**Affiliations:** 1 Service d'orthopédie-traumatologie, établissement public hospitalier de Sikasso, Mali; 2 Service d'orthopédie-traumatologie, établissement public hospitalier de Mopti, Mali; 3 Service d'orthopédie-traumatologie, Centre National Hospitalier Universitaire Hubert Koutougou MAGA de Cotonou (CNHU-HKM), Bénin

**Keywords:** *Madurella*, Mycétome, Actinomycétome, Eumycétome, Traitement médico-chirurgical, Hôpital, Mopti, Mali, Afrique subsaharienne, *Madurella*, Mycetoma, Actinomycetoma, Eumycetoma, Medico-surgical treatment, Hospital, Mopti, Mali, Sub-Saharan Africa

## Abstract

**Objectif:**

L'objectif de cette étude était de décrire les aspects épidémiologiques, cliniques et thérapeutiques des mycétomes.

**Matériel et méthodes:**

Il s'agit d'une étude rétrospective incluant les patients traités pour mycétome de janvier 2016 à décembre 2018 dont deux ans de recrutement et un an de suivi (2019) à l'hôpital de deuxième niveau (Sominé Dolo de Mopti). Elle a concerné 19 patients hospitalisés et traités dans le service de chirurgie.

**Résultats:**

L’étude portait sur 11 hommes et huit femmes. L’âge moyen de nos patients était de 38 ans, avec des extrêmes de 15 - 70 ans. Le délai médian de consultation était de dix ans après le début des symptômes (1 - 40 ans). Les éleveurs et les cultivateurs étaient concernés dans huit et sept cas respectivement’ avec la notion de traumatisme dans 14 cas. Le pied était la zone de prédilection dans 13 cas. Une atteinte osseuse et ostéoarticulaire était notée chez 12 des patients. Des grains noirs étaient présents dans 16 cas attribués à *Madurella* sp. Nous avons réalisé 12 amputations, six exérèses carcinologiques et des soins locaux pour un patient.

**Conclusion:**

Les mycétomes doivent être évoqués et diagnostiqués à un stade précoce chez les sujets prédisposés particulièrement les agriculteurs et les éleveurs. La prévention est d'une grande importance, elle repose sur la désinfection des plaies et le port de chaussures protectrices.

## Abstract (en)

**Objective:**

The objective of this study was to describe the epidemiological and clinical aspects as well as the therapeutic methods of mycetomical lesions.

**Material and Methods:**

This was a longitudinal retrospective study, which included all patients treated for mycetoma from January 2016 to December 2018 including two years of recruitment and one year of monitoring (2019). The study concerned 19 patients who were hospitalized and treated in the department of surgery.

**Results:**

Patients represented 2.3% of hospitalizations and consisted of 11 males and 8 females with an average age of 38 years with extremes of 15 - 70 years, and an average time between the onset of symptoms and presentation to the hospital of 10 years (range 1 - 40 years). Eight livestock breeders and seven farmers were concerned, 14 of whom have started the disease after trauma. The foot was involved in 13 patients. Twelve suffered from osteoarticular lesions. Black grains were present in 16 cases attributed to *Madurella* sp. We performed 12 amputations, six carcinological ablation to which specific local treatments were added (thin skin graft in two patients, fasciocutaneous flap in one patient and directed healing in the others) and local treatment in the last case.

**Conclusion:**

Mycetoma should be discussed and diagnosed at an early stage in predisposed patients particularly in farmers and breeders. Prevention is necessary; it is based on wound disinfection and wearing safety shoes.

## Introduction

Les mycétomes constituent des pseudotumeurs inflammatoires indolentes et déformantes souvent polyfistulisées dues à des champignons (eumycétome) ou à des bactéries aérobies (actinomycétome) [19].

Ils affectent surtout le sujet jeune, de sexe masculin, travailleur manuel, tels que les agriculteurs et éleveurs, issu des zones tropicales arides [11, 14, 19].

La notion de traumatisme antérieur est retrouvée dans 60 % des cas et le siège de prédilection est le pied [23].

L’évolution lente et progressive des lésions des téguments et des parties molles finit souvent par une atteinte secondaire articulaire et du squelette sous-jacent. Cette atteinte secondaire entraîne une complication fréquente et redoutable conditionnant le pronostic fonctionnel et esthétique [4].

La région de Mopti est située au centre du Mali. Elle est divisée en deux grandes zones agro-écologiques (une zone exondée et une zone inondée).

Cette situation géographique particulière de la région représente un grand handicap pour les références/évacuations à partir de la zone inondée pendant la crue du fleuve à l'hivernage (juin, juillet, août et septembre).

Selon les résultats du projet santé population hydraulique rurale (PSPHR), il s'agit d'une région pauvre avec un taux d'incidence de pauvreté supérieur à 76 % au sein de la population de la région de Mopti (dont environ 38 % de pauvres et 38 % de très pauvres). Elle est éligible à la mise en œuvre du programme de développement sanitaire et social (PRODESS). L'agriculture, la pêche et l’élevage constituent les principales activités, elles occupent près de 90 % de la population. La pyramide sanitaire de la région de Mopti a fortement évolué à la faveur des différentes péripéties de la mise en œuvre de la politique nationale de santé et de population. Elle se compose ainsi de nos jours: un hôpital régional, huit centres de santé de référence et 128 centres de santé communautaire fonctionnels.

**Figure 1:**
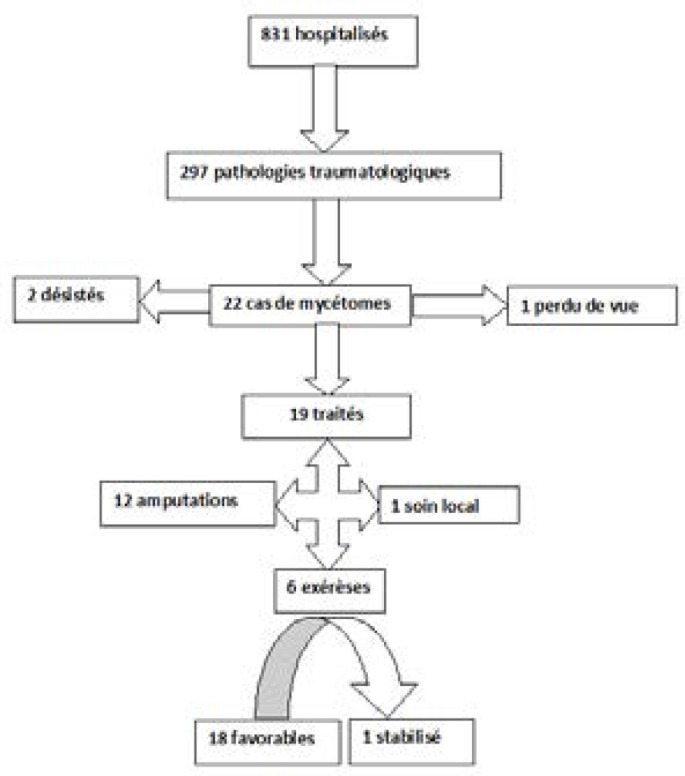
Diagramme de flux de l’étude Flow chart of the study

La recrudescence du nombre d'amputations liées au retard diagnostic et thérapeutique nous a incité à réaliser cette étude avec comme objectifs de décrire les aspects épidémio-cliniques et radiologiques des lésions mycétomiques et présenter les méthodes thérapeutiques (Fig. 1).

## Matériel et méthodes

Il s'agit d'une étude rétrospective incluant tous les patients provenant des régions du nord (Kidal, Tombouctou, Gao et Mopti) et traités pour mycétome pendant la période d’étude (janvier 2016 à décembre 2018) comprenant deux ans de recrutement et un an de suivi. Elle a concerné les patients hospitalisés et traités dans le service de chirurgie qui regroupe les unités chirurgicales dont l'orthopédie-traumatologie.

L'hôpital Sominé Dolo de Mopti est l'unique structure médico-chirurgicale de 2^e^ niveau de cette 5^e^ région administrative du Mali.

Il est situé dans la zone administrative de Sévaré au bord de la route nationale 6 (RN6).

Il a pour missions d'assurer:

- les soins curatifs de 2^e^ référence et la prise en charge des urgences;
- la formation (contribution à la formation initiale des élèves et étudiants et la formation continue des personnels médicaux et paramédicaux);
- la recherche dans le domaine de la santé.

L'hôpital est composé de services eux-mêmes subdivisés en unités.

Ainsi, le service de chirurgie regroupe les unités d'orthopédie-traumatologie, de chirurgie générale, d'urologie, d'ORL et de chirurgie maxillo-faciale.

Les patients refusant le traitement ou perdus de vue ont été exclus de l’étude.

Le diagnostic était clinique et paraclinique. Il était retenu en présence d'une polyfistulisation cutanée, laissant sourdre du pus et des grains à l'examen direct, d'une infiltration œdémateuse et de déformation parfois associées à une impotence fonctionnelle et une douleur évocatrice d'une surinfection ou d'une atteinte osseuse évoluée. Sur la radiographie standard des géodes et des lyses osseuses confirmaient une atteinte osseuse (Fig. 3). Une biopsie a été pratiquée dans notre structure pour examen anatomopathologique. La pièce était fixée dans du formol puis envoyée au CHU du Point-G à Bamako. Les résultats étaient disponibles dans un délai de sept à dix jours. Nous n'avons pas procédé à des cultures pour l'identification des germes.

## Résultats

**Figure 2:**
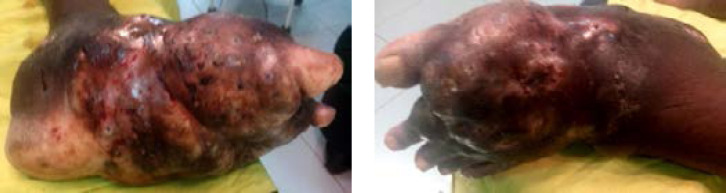
Eumycétome pied gauche évoluant depuis 20 ans chez un homme, 62 ans, cultivateur Eumycetoma of the left foot evolving for 20 years in a 62-year-old male farmer

Sur 831 patients hospitalisés durant la période d’étude, nous avons colligé 19 cas de mycétome soit une prévalence de 2,3 %. Deux patients ont refusé le traitement et un a été perdu de vue après la première consultation (Fig. 2).

**Figure 3:**
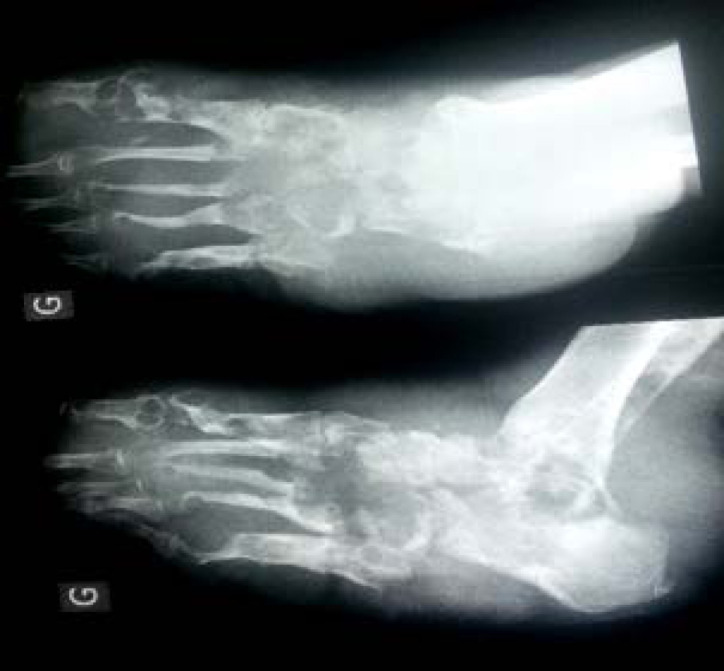
Radiographie objectivant une atteinte ostéoarticulaire avec lyse osseuse d'un eumycétome pied gauche évoluant depuis 20 ans chez un homme, 62 ans, cultivateur X-ray showing osteoarticular involvement with bone lysis of a left foot eumycetoma evolving for 20 years in a 62-year-old male farmer

L’échantillon était composé de 11 hommes et huit femmes. L’âge moyen de nos patients était de 38 ans avec des extrêmes de 15 ans et de 70 ans à la première consultation. Le délai médian de consultation était de dix ans après le début des symptômes (1 - 40 ans).

Les patients provenaient de Tombouctou dans neuf cas, Mopti dans huit cas et Gao dans deux cas. Les éleveurs étaient concernés dans huit cas, les cultivateurs dans sept cas. Les autres cas étaient: comptable, chauffeur, ménagère et commerçant représentaient chacun un cas.

Le mécanisme traumatique était incriminé dans 14 cas, piqûre d'arbre épineux (huit cas), arête de poisson (six cas) et il était inconnu dans cinq cas.

Les principales localisations étaient les pieds dans 13 cas, les jambes et les mains dans deux cas chacune, le tronc et le genou dans un cas chacun. Pour le tronc il s'agissait du flanc droit traité comme un zona. Les lésions étaient profondes dans 12 cas et superficielles dans sept cas.

Cliniquement une confirmation de la nature mycétomique de l'affection était révélée par la triade tuméfaction, fistule et émission de grains caractéristiques: noirs ou blancs visibles à l’œil nu chez tous nos patients. La biopsie a été réalisée chez tous nos patients et toutes les pièces ont été envoyées pour l'anatomopathologie au centre hospitalo-universitaire du Point-G.

L'examen histologique a diagnostiqué 16 cas de mycétomes à *Madurella mycetomis* et trois cas *d'Actinomadura madurae.*

Sur le plan radiologique nous avons noté une atteinte osseuse et ostéoarticulaire dans 12 cas et six cas sans atteinte osseuse. L’échographie abdominale de la lésion du flanc n'a pas révélé de complication.

Au plan thérapeutique, dix de nos patients avaient déjà reçu un traitement traditionnel (fumigations, scarification et application de poudre noire), six ont eu un traitement médical et trois un traitement médicochirurgical.

Devant la rechute, l'extension importante des lésions et l'atteinte ostéoarticulaire, la chirurgie radicale a été inévitable. L'indication d'une amputation d'emblée a été posée chez les patients qui présentaient des mycétomes à longue durée d’évolution (entre 4 et 40 ans) avec une importante extension ostéoarticulaire.

L'amputation a été pratiquée chez 12 patients, dont sept avait déjà a été faites dans un centre de santé de référence par un médecin généraliste. Elle a été réalisée dans neuf cas à la jambe, dans deux cas à la cuisse et un cas au poignet.

Nous avons réalisé six exérèses carcinologiques de masses mycétomiques parmi lesquelles trois patients ont bénéficié des gestes complémentaires: à savoir une greffe de peau mince chez deux patients ayant intéressé le pied et un lambeau fascio-cutané au niveau de la cheville. Les trois autres patients ont fait l'objet d'une cicatrisation dirigée. Nous avons eu des résultats satisfaisants avec cicatrisation complète de la plaie opératoire et conservation de la mobilité articulaire du pied et de la cheville; aucune récidive n'a été constatée. Le dernier patient a bénéficié des soins locaux (Tableau I).

**Tableau I:** Caractéristiques de la série Characteristics of the series

Variables	Effectifs
**Sexe**	
homme	11
femme	8
**Provenance**	
Tombouctou	9
Mopti	8
Gao	2
**Activités / profession**	
éleveurs	8
cultivateurs	7
autres	4
**Traumatisme initial**	
piqûre végétale	8
arête de poisson	6
inconnu	5
**Siège anatomique**	
pieds	13
jambes	2
main	2
genou	1
abdomen	1

Le traitement médical était à base de kétoconazole ou itraconazole 200 mg associé au triméthoprime-sulfaméthoxazole 960 mg en deux prises par jour pendant deux mois puis une prise journalière pendant dix mois selon l’état de gravité des lésions, de la durée d’évolution et surtout des récidives. Il était instauré avant l'intervention.

Tous nos patients ont été revus pendant l'année de suivi avec un intervalle de trois mois et dix-sept patients ont été évalués avec un recul moyen de 23 mois (extrême 5 et 36 mois).

Nous avons enregistré un cas d'infection du moignon, pas de récidive après 12 mois de suivi.

L’évolution a été favorable chez 18 patients et stable chez le dernier. Le suivi clinique des patients était difficile à cause de la distance des localités, de la période hivernale des zones inondées et les moyens de transport difficiles.

## Discussion

La littérature confirme le long délai de consultation des patients ayant un mycétome [23].

Les lésions siègent majoritairement au pied [1,3,16,22] comme le confirme notre étude. Cependant, des localisations extrapodales ont été décrites dans la littérature, telles que la région du tronc, les mains ou les genoux [12, 15, 26]. Les agriculteurs et les éleveurs sont les plus exposés à cette pathologie [8, 10, 17]. Les mycétomes ont une tendance à s’étendre en superficie même en profondeur envahissant progressivement tous les tissus avoisinants dont l'os et les articulations [9, 20]. Ils évoluent lentement et restent indolores pendant de nombreux mois, voire d'années [21], ce qui explique les consultations tardives [3, 4].

L'examen direct oriente le clinicien vers une étiologie fongique (eumycétome) si la couleur est noire et vers une étiologie bactérienne si la couleur est rouge [12].

Les grains blancs sont rencontrés aussi bien au cours des mycétomes fongiques qu'actinomycosiques [5, 24].

Le traitement des mycétomes dépend de leur étiologie. Il n'existe pas de consensus bien établi, mais le traitement doit être poursuivi longtemps [16]. Différentes thérapeutiques peuvent être proposées en cas d'actinomycétomes, telles que la dapsone, les sulfamides, le triméthoprime-sulfaméthoxazole ou la rifampicine et le traitement chirurgical est réservé aux formes résistantes à l'antibiothérapie et évoluées [11, 19].

Les eumycétomes nécessitent l'association d'antifongiques et de traitements chirurgicaux (le plus souvent à type d'amputation en cas de forme évoluée) et le kétoconazole ou l'itraconazole sont indiqués en première intention [16].

Dans les eumycétomes, la chirurgie encadrée par des antifongiques garde une place de choix, soit sur les lésions débutantes, soit sur celles très évoluées [2, 6, 7]. Dans les cas comportant des lésions très limitées, encapsulées ou enkystées, l'exérèse est facile, le risque de récidive très faible. À l'inverse, dans les cas avancés, on ne peut proposer qu'une amputation [13, 25].

L'indication d'exérèse a été posée devant l'absence d'une extension en superficie et en profondeur des tissus avoisinants et la taille plus ou moins limitée de la tumeur. Le geste chirurgical consiste en une exérèse carcinologique emportant en monobloc c'est-à-dire passant en zone saine de 2-4 cm. Cette chirurgie peut poser de difficiles problèmes de reconstruction cutanée, musculaire pouvant justifier des greffes ou des lambeaux.

Les récidives après exérèse s'expliquent par le fait que l'exérèse initiale doit être carcinologique. Dans le cas contraire, quelques grains persistent après intervention provoquant ainsi la reprise du processus infectieux [13, 22].

Des auteurs ont proposé un traitement médical à base d'antifongique en vue d'encadrer le traitement chirurgical [18, 22]. L'objectif est de mieux circonscrire les lésions en vue de faciliter la chirurgie et d’éviter les récidives.

Parmi nos patients amputés et évalués, nous n'avons pas observé de récidive. Cependant aucun de nos patients n'a pu bénéficier d'appareillage en raison de l'absence d'un centre d'appareillage dans la région de Mopti et de moyens financiers pour se rendre à Bamako.

Le suivi de ces patients est d'abord clinique et doit durer plusieurs années. Ce qui est difficilement réalisable du fait que les patients sont perdus de vue, de l’état défectueux des routes, des zones inondées pendant la saison pluvieuse et de l'insécurité grandissante dans le centre du Mali en raison du positionnement des rebelles et des engins explosifs placés le long des routes.

## Conclusion

Quelle que soit la méthode thérapeutique, les mycétomes doivent être évoqués et diagnostiqués à un stade précoce chez les sujets exposés particulièrement les agriculteurs et les éleveurs. La prévention est d'un grand intérêt, elle repose sur la désinfection des plaies et le port de chaussures protectrices.

L'information, la sensibilisation des usagers et du personnel de santé devraient contribuer à une meilleure fréquentation des structures sanitaires en cas de pseudotumeur du pied, fistulisée ou non. La formation continue du personnel de santé sur les maladies tropicales négligées permettrait également de réduire les complications et les handicaps liés aux amputations.

Les auteurs recommandent aux pouvoirs publics et aux institutions nationales des campagnes d’éducation et de chirurgie de mycétome dans les régions endémiques.

## Liens d'intérêts

Les auteurs déclarent ne pas avoir de liens d'intérêt.

## Source de financement

Il s'agit d'une étude à financement personnel.

## Contribution des auteurs

Terna TRAORE et Mathias DIASSANA: ont contribué à la conception de l’étude

Terna TRAORE et Layes TOURE: ont contribué à la rédaction du manuscrit

Aristide HANS-MOEVI: a révisé et validé le protocole de l’étude

Boubacar S COULIBALY et Mamadou NIANG: ont contribué à la collecte des données et à l'analyse

Terna TRAORE et Emmanuel BALLO: ont contribué au suivi des patients

Aristide HANS-MOEVI: a lu et approuvé la version finale du manuscrit
